# miR-9-5p, miR-675-5p and miR-138-5p Damages the Strontium and LRP5-Mediated Skeletal Cell Proliferation, Differentiation, and Adhesion

**DOI:** 10.3390/ijms17020236

**Published:** 2016-02-15

**Authors:** Tianhao Sun, Frankie Leung, William W. Lu

**Affiliations:** 1 Department of Orthopaedics and Traumatology, Li Ka Shing Faculty of Medicine, The University of Hong Kong, Hong Kong, China; sunmishu@126.com; 2 Shenzhen Key Laboratory for Innovative Technology, The University of Hong Kong Shenzhen Hospital, Shenzhen 518000, China; 3 Shenzhen Institutes of Advanced Technology, Chinese Academy of Science, Shenzhen 518000, China

**Keywords:** microRNA or miRNA, bone, strontium, cell proliferation, differentiation, adhesion and apoptosis, target, LRP5

## Abstract

This study was designed to evaluate the effects of strontium on the expression levels of microRNAs (miRNAs) and to explore their effects on skeletal cell proliferation, differentiation, adhesion, and apoptosis. The targets of these miRNAs were also studied. Molecular cloning, cell proliferation assay, cell apoptosis assay, quantitative real-time PCR, and luciferase reporter assay were used. Strontium altered the expression levels of miRNAs *in vitro* and *in vivo*. miR-9-5p, miR-675-5p, and miR-138-5p impaired skeletal cell proliferation, cell differentiation and cell adhesion. miR-9-5p and miR-675-5p induced MC3T3-E1 cell apoptosis more specifically than miR-138-5p. miR-9-5p, miR-675-5p, and miR-138-5p targeted glycogen synthase kinase 3 β (GSK3β), ATPase Aminophospholipid Transporter Class I Type 8A Member 2 (ATP8A2), and Eukaryotic Translation Initiation Factor 4E Binding Protein 1 (EIF4EBP1), respectively. Low-density lipoprotein receptor-related protein 5 (LRP5) played a positive role in skeletal development. miR-9-5p, miR-675-5p, and miR-138-5p damage strontium and LRP5-mediated skeletal cell proliferation, differentiation, and adhesion, and induce cell apoptosis by targeting GSK3β, ATP8A2, and EIF4EBP1, respectively.

## 1. Introduction

Strontium ranelate is a drug used for treating osteoporosis. The effects of strontium result in increased bone formation and decreased bone resorption [1]. Our previous studies showed that strontium promoted osteogenic differentiation of mesenchymal stem cells [2]. However, the exact mechanism of the effects of strontium ranelate remains unclear.

The miRNAs play vital roles in bone formation, so they may be used in the diagnosis and treatment of many diseases such as osteoporosis. For instance, miR-138 inhibits the osteogenic differentiation of mesenchymal stem cells [3]. miR-370 remarkably attenuates BMP-2-induced pre-osteoblast differentiation [4]. miRNAs inhibit gene expression through binding to the 3′-untranslated region (3′UTR) of target mRNAs. Multiple studies have demonstrated that miRNAs regulate most biological processes of cells, including proliferation, differentiation, and apoptosis [5]. Severe diseases can be attributed to abnormal expression of miRNA. Though there are many studies in the relationship between the miRNAs and bone remodeling, the studies for the strontium regulating the miRNAs are very few.

We and other investigators found that the expression levels of β-cantenin increased significantly after strontium treatment [6], and the Wnt signaling pathways were activated by strontium. Wnt signaling pathways play important roles in bone formation. LRP5 is a co-receptor of the Wnt signaling pathways. Our previous study indicated that strontium could increase the mRNA levels of LRP5 in wild-type mice [6]. Sclerostin has anti-anabolic effects on skeletal development and inhibits osteoblast differentiation and bone formation [7]. Sclerostin has been identified as binding to LRP5 and inhibiting the Wnt signaling pathways [8]. The inhibition of the Wnt signaling pathways results in decreased bone formation [9]. Transfection of a hairy/enhancer-of-split related with YRPW motif protein 1 (Hey1) expression plasmid reversed the upregulation of differentiation markers in the MC3T3-E1 cells [10]. This revealed the importance of Hey1 as a potent negative regulator of osteogenesis in regulating osteoblastic differentiation. The studies have showed that Hey1 negatively influences osteoblastic differentiation and inhibition of Hey1 enhanced the osteoblastic differentiation. Mothers against decapentaplegic homolog 6 (Smad6) acts as a negative regulator of bone morphogenetic proteins. It is demonstrated that Mothers against decapentaplegic homolog 7 (Smad7), as well as Smad6, inhibits proliferation and differentiation of osteoblastic cells [11].

In this study, we detected the expression levels of miRNAs affected by the strontium and explored the roles of these miRNAs in skeletal development. In addition, we studied the potential targets of three important miRNAs and the roles LRP5 played in bone formation.

## 2. Results

### 2.1. Strontium Altered the Expression Levels of miRNAs in Vitro and in Vivo

**Figure 1:**
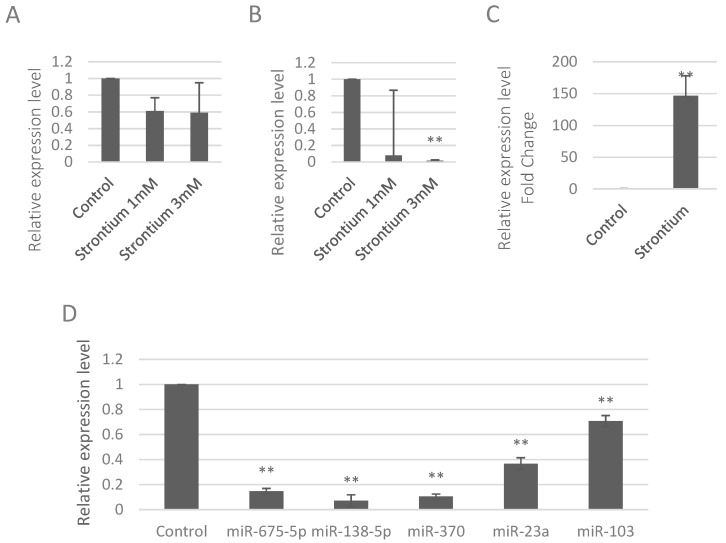
Strontium altered the expression levels of miRNA *in vitro* and *in vivo*. (**A**) Expression levels of miR-9-5p in MC3T3-E1 cells upon strontium ranelate treatment; (**B**) Expression levels of miR142-3p in MC3T3-E1 cells upon strontium ranelate treatment; (**C**) Expression levels of ALP in the bone of the mice upon strontium treatment; (**D**) Expression levels of miRNAs in the bone of mice upon strontium treatment. ** *p* < 0.01.

MC3T3-E1 cells (4 × 10^5^ cells/well) in the six-well plates were treated with strontium ranelate six hours after seeding. We detected the miRNAs that were related to bone formation according to previous studies [12,13,14,15,16,17,18]. Results showed the down-regulation of miR-9-5p upon strontium ranelate treatment but there were no significant changes (Figure 1A). We also found the expression of miR-142-3p decreased after the strontium treatment (Figure 1B). Additionally we detected other miRNAs including miR-675-5p, miR-138-5p, miR-27b, miR-140, miR-141, and miR-103 but there were no changes in the expression levels of these miRNAs (Figure S1).

To further study the effects of strontium on expression levels of miRNAs *in vivo*, we treated the mice with strontium ranelate. To verify the positive effects of strontium on bone formation of these mice, we detected the alkaline phosphatase (ALP), a byproduct of osteoblast activity, and it increased if there was bone formation occurring. Results showed that ALP increased significantly with the fold change of 146.4 in the bone of the mice treated with strontium (Figure 1C), suggesting that strontium promoted bone formation *in vivo*. Then, we detected the levels of miRNAs in the bone of mice. Results showed that the expression levels of miR-675-5p, miR-138-5p, miR-370, miR-23a, and miR-103 were significantly inhibited by strontium ranelate (Figure 1D).

### 2.2. miR-9-5p, miR-675-5p, and miR-138-5p Reduced Skeletal Cell Count and Cell Proliferation

Since our data showed strontium inhibited miR-9-5p and miR-142-3p *in vitro*, we further studied previous reports of these two miRNAs. miR-9 dysregulation is implicated in a variety of human diseases, but its role remains contradictory [19,20]. In addition, its roles in bone formation were not well studied. We also further studies the five decreased miRNAs *in vivo*. miR-138-5p was the most significantly inhibited miRNA among these five miRNAs, and there were few studies of the influences of miR-675-5p on skeletal development. Thus, we further studied miR-9-5p, miR-675-5p, and miR-138-5p.

**Figure 2:**
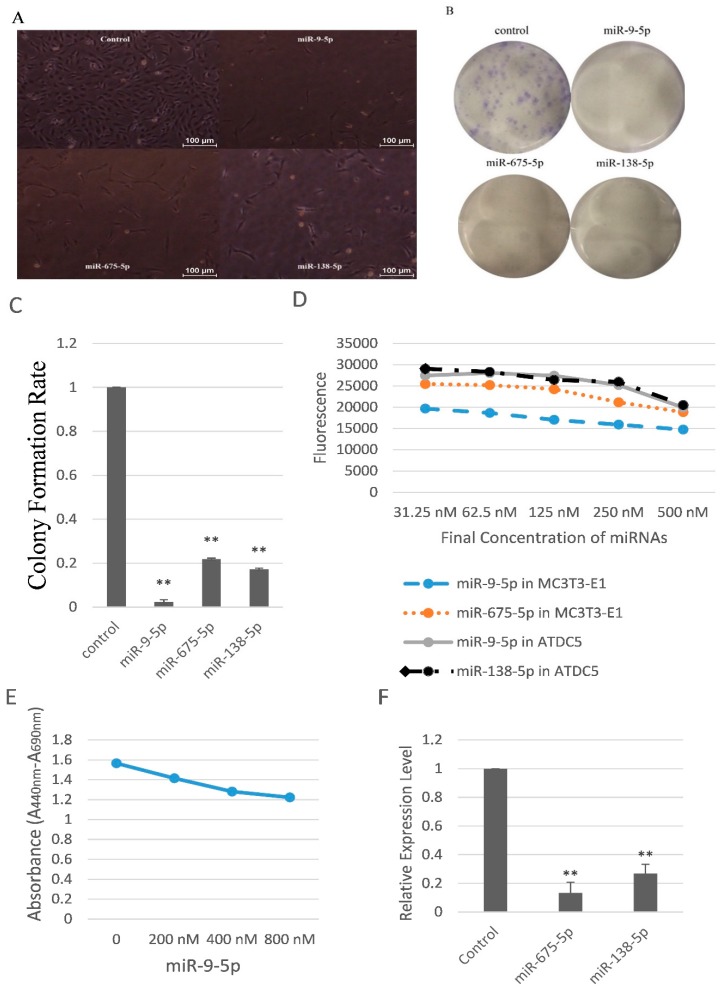
miR-9-5p, miR-675-5p, and miR-138-5p reduced skeletal cell count and cell proliferation. (**A**) Representative photos of MC3T3-E1 cells transfected with miR-9-5p, miR-675-5p, and miR-138-5p; (**B**) Representative pictures of clonogenic assay with miR-9-5p, miR-675-5p, and miR-138-5p; (**C**) The clonogenic potential of MC3T3-E1 transfected with miR-9-5p, miR-675-5p, and miR-138-5p; (**D**) Fluorescence changes of MC3T3-E1 transfected with miR-9-5p and miR-675-5p, and ATDC5 transfected with miR-9-5p and miR-138-5p; (**E**) Determination of the cytotoxic activity of miR-9-5p on MC3T3-E1 cells; (**F**) Expression levels of PCNA in MC3T3-E1 cells upon transfection of miR-675-5p and miR-138-5p ** *p* < 0.01. *n* = 3/group.

First of all, we studied the influences on cell count of these three miRNAs. We seeded the same numbers of MC3T3-E1 cells (5 × 10^4^ cells/well) in six-well plates. The cells were transfected with miR-9-5p, miR-675-5p, and miR-138-5p mimics (40 nM). Every group contained triplicate wells. Photos were taken four days after transfection. The numbers of cells treated with miR-9-5p, miR-675-5p, and miR-138-5p mimics were apparently less than the control group (Figure 2A), indicating that miR-9-5p, miR-675-5p, and miR-138-5p, especially miR-9-5p, reduced cell count.

To further investigate mechanisms underlying miR-dependent changes in cell count, we conducted a series of cell proliferation assays. Firstly, clonogenic assay results revealed that clonogenic survival were decreased following overexpression of miR-9-5p, miR-675-5p, and miR-138-5p in the MC3T3-E1 cells (Figure 2B,C). Secondly, we tested the cell viability of MC3T3-E1 and ATDC5 cells transfected with serial dilution of miRNAs by using resazurin measurement. With the increase of the concentration of miR-9-5p, the fluorescence decreased (Figure 2D), indicating that miR-9-5p damaged viability of these two kinds of cells. In addition, miR-675-5p decreased the MC3T3-E1 cell viability while miR-138-5p decreased the ATDC5 cell viability (Figure 2D). Thirdly, WST-1-based colorimetric assay showed that miR-9-5p had cell cytotoxicity and inhibited MC3T3-E1 cell proliferation in a dose-dependent manner (Figure 2E). Fourthly, we detected the levels of proliferating cell nuclear antigen (PCNA), an important proliferation marker. Results showed that miR-675-5p and miR-138-5p significantly decreased PCNA in MC3T3-E1 cells (Figure 2F). We also found that miR-9-5p significantly (*p* < 0.01) reduced the levels of Cyclin A, Cyclin D1, and Cyclin E in ATDC5 cells, and miR-675-5p significantly (*p* < 0.01) reduced the Cyclin E in MC3T3-E1 and Cyclin D1 in ATDC5 cells (data not shown).

Taken together, these data strongly suggested that miR-9-5p, miR-675-5p, and miR-138-5p reduced skeletal cell proliferation.

### 2.3. miR-9-5p, miR-675-5p, and miR-138-5p Impaired Skeletal Cell Differentiation and Cell Adhesion

**Figure 3:**
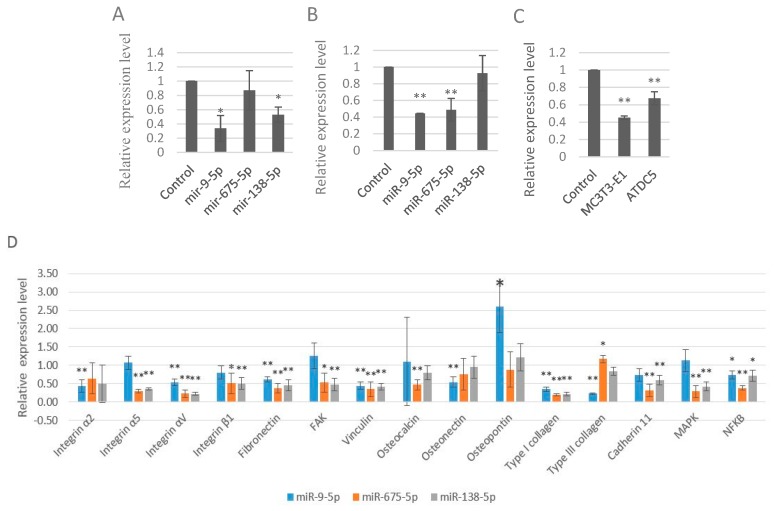
miR-9-5p, miR-675-5p, and miR-138-5p impaired skeletal cell differentiation and cell adhesion. (**A**) Expression levels of LRP5 in MC3T3-E1 cells upon transfection of miR-9-5p, miR-675-5p, and miR-138-5p; (**B**) Expression levels of RUNX2 in MC3T3-E1 cells upon transfection of miR-9-5p, miR-675-5p, and miR-138-5p, respectively; (**C**) Expression levels of COL1A1 upon transfection of miR-9-5p (5 nM); (**D**) Expression levels of 15 cell adhesion markers in MC3T3-E1 cells upon transfection of miR-9-5p, miR-675-5p, and miR-138-5p. * *p* < 0.05. ** *p* < 0.01. *n* = 3/group.

We further studied the roles of these three miRNAs played in the expression levels of LRP5 and Runt-related transcription factor 2 (RUNX2). RUNX2 is a key transcription factor associated with osteoblast differentiation. Results showed all these three miRNAs inhibited the expression levels of LRP5 and RUNX2 (Figure 3A,B). In addition, miR-9-5p inhibited the LRP5 and RUNX2 more significantly than the other two miRNAs. To further confirm these results, we used another cell line ATDC5, a model for skeletal development. Results were similar with that of MC3TC-E1cells (data not shown). More specifically, miR-9-5p, miR-675-5p, and miR-138-5p inhibited the expression of RUNX2 in ATDC5 cells. In addition, miR-9-5p significantly (*p* < 0.01) inhibited the levels of RUNX2 in ATDC5 cells (data not shown). Since all these data suggested that miR-9-5p more adversely impaired the skeletal development, we detected another biomarker Collagen, type I, α 1 (COL1A1), a protein that strengthens and supports bone. Results showed that miR-9-5p significantly inhibited the levels of COL1A1 in both MC3TC-E1 and ATDC5 cells (Figure 3C).

We examined total 15 cell adhesion markers including four integrins (Integrin α2, Integrin α5, Integrin αV, and Integrin β1), one extracellular matrix component (Fibronectin), 2 focal adhesion markers (FAK and Vinculin), three osteoblast specific markers (Osteocalcin, Osteonectin and Osteopontin), two collagens (Type I collagen and Type III collagen), Cadherin 11, Mitogen Activated Protein Kinase (MAPK), and Nuclear Factor κB (NFκB) according to previous studies [21]. The results of these three miRNAs regulating the 15 cell adhesion markers in MC3T3-E1 cells were listed in the Figure 3D and Table S1. miR-9-5p, miR-675-5p, and miR-138-5p generally decreased these 15 cell adhesion markers with a few exceptions (Figure 3D), suggesting that these three miRNAs decreased skeletal cell adhesion.

Taken together, these data strongly suggested that miR-9-5p, miR-675-5p, and miR-138-5p, impaired the skeletal cell differentiation and cell adhesion.

### 2.4. miR-9-5p and miR-675-5p Induced MC3T3-E1 Cell Apoptosis More Specifically than miR-138-5p

**Figure 4:**
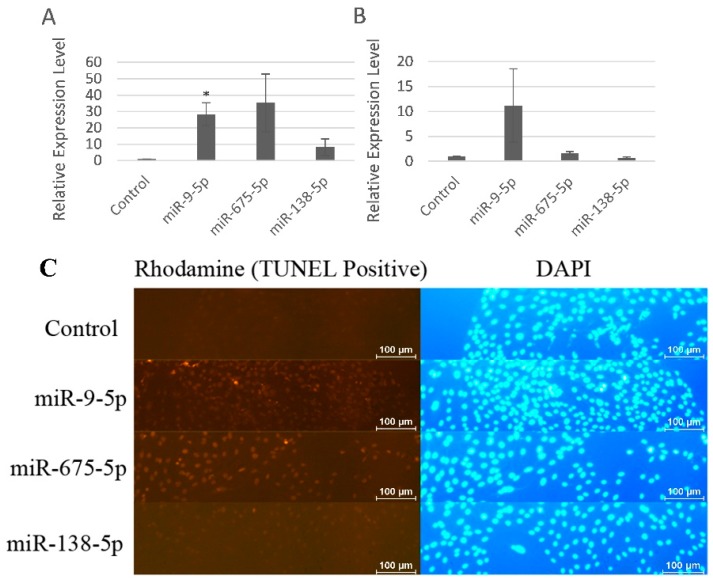
miR-9-5p and miR-675-5p induced MC3T3-E1 cell apoptosis more specifically than miR-138-5p. (**A**) Expression levels of FasL in MC3T3-E1 cells upon transfection of miR-9-5p, miR-675-5p, and miR-138-5p; (**B**) Expression levels of p53 in MC3T3-E1 cells upon transfection of miR-9-5p, miR-675-5p, and miR-138-5p, respectively; (**C**) TUNEL cell apoptosis assay of miR-9-5p, miR-675-5p, and miR-138-5p. * *p* < 0.05. *n* = 2/group.

miR-9-5p significantly increased the levels of pro-apoptotic regulator Fas ligand (FasL) with fold change of 28.3 and miR-675-5p increased the levels of FasL with fold change of 35.3, while miR-138-5p slightly increased the levels of FasL (Figure 4A). We also detected another classical pro-apoptotic regulator p53, and the results were similar. miR-9-5p induced MC3T3-E1 cell apoptosis more specifically than miR-138-5p (Figure 4B). TUNEL cell apoptosis assay showed that miR-9-5p and miR-675-5p induced cell apoptosis while miR-138-5p slightly induced apoptosis (Figure 4C). In other words, cell apoptosis seen by miR-9-5p and miR-675-5p overexpression were more specific than that of miR-138-5p.

### 2.5. miR-9-5p, miR-675-5p, and miR-138-5p Targeted GSK3β, ATP8A2, and EIF4EBP1

**Figure 5:**
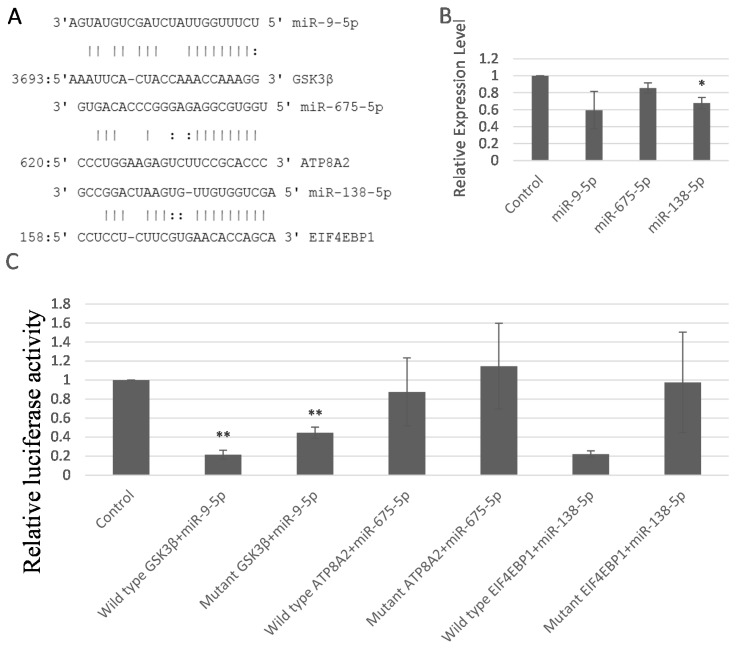
miR-9-5p, miR-675-5p and miR-138-5p targeted GSK3β, ATP8A2, and EIF4EBP1, respectively. (**A**) The predicted miR-9-5p, miR-675-5p, and miR-138-5p targeting sequence located in the 3’UTR of GSK3β, ATP8A2, and EIF4EBP1; (**B**) The mRNA level of GSK3β, ATP8A2, and EIF4EBP1 in MC3T3-E1 cells transfected with miR-9-5p, miR-675-5p, and miR-138-5p mimics; (**C**) The relative luciferase activity of MC3T3-E1 cells transfected with miR-9-5p, miR-675-5p, and miR-138-5p mimics (20 nM for miR-675-5p and miR-138-5p, and 10 nM for miR-9-5p). * *p* < 0.05. ** *p* < 0.01. *n* = 3/group.

To further study the downstream targets of these miRNAs, we used the miRNA target prediction software to seek for the potential targets. The genes GSK3β, ATP8A2, and EIF4EBP1 were predicted to be the targets of miR-9-5p, miR-675-5p, and miR-138-5p, respectively. Figure 5A showed the predicted miR-9-5p, miR-675-5p, and miR-138-5p targeting sequence located in the 3’UTR of GSK3β, ATP8A2, and EIF4EBP1, respectively.

Then we assessed whether overexpression of miR-9-5p, miR-675-5p, and miR-138-5p suppressed the expression of GSK3β, ATP8A2, and EIF4EBP1. Forced expression of miR-9-5p, miR-675-5p, and miR-138-5p inhibited the mRNA level of GSK3β, ATP8A2, and EIF4EBP1, respectively, in the MC3T3-E1 cell (Figure 5B). There were concordant results in another cell line, ATDC5 (data not shown).

To confirm the regulation of GSK3β, ATP8A2, and EIF4EBP1 by miR-9-5p, miR-675-5p, and miR-138-5p, we cloned and inserted the wild-type and mutant fragments of the 3’UTR of these three genes containing the binding sites of miR-9-5p, miR-675-5p, and miR-138-5p into the luciferase vector. Luciferase–3’UTR plasmids were co-transfected into MC3T3-E1 cells with or without miRNA mimics, followed by Dual-Light luminescent reporter gene assay. Transfection of miR-9-5p, miR-675-5p and miR-138-5p mimics resulted in a reduction in luciferase activity in MC3T3-E1 cells transfected with wild type Luciferase–3’UTR plasmids (Figure 5C), while the inhibition of luciferase activity by these three miRNAs was abrogated in MC3T3-E1 cells transfected with mutant plasmids. miR-9-5p, miR-675-5p, and miR-138-5p inhibited their wild-type targets more significantly than mutant ones (Figure 5C), so resistance of mutant luciferase vectors to miRNAs confirmed the specific targeting.

Taken together, these results strongly suggested that miR-9-5p, miR-675-5p, and miR-138-5p decreased GSK3β, ATP8A2, and EIF4EBP1 by directly binding to their 3’UTR.

### 2.6. LRP5 Played Positive Roles in Skeletal Development

**Figure 6:**
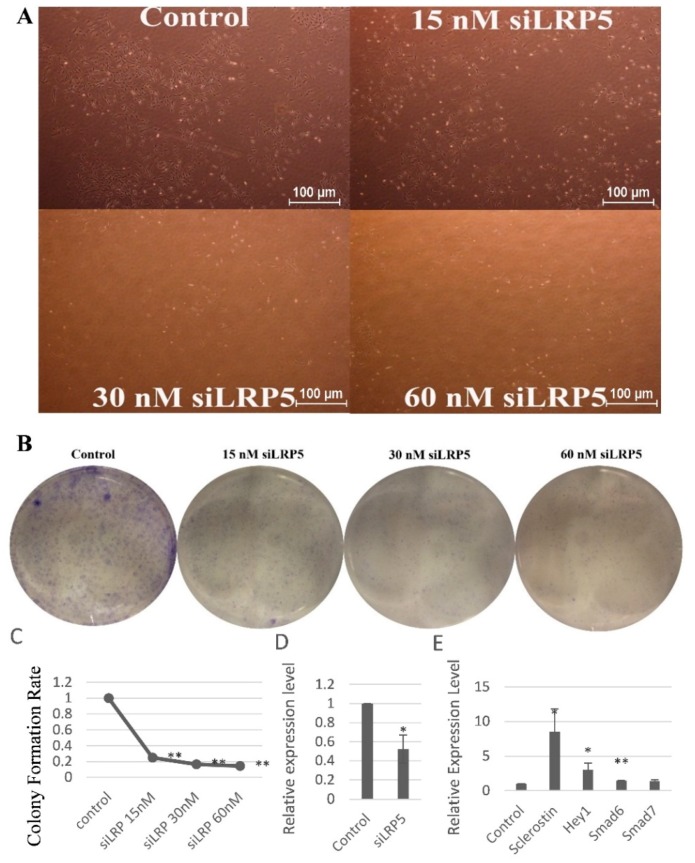
LRP5 played positive roles in the skeletal development. (**A**) Representative photos of MC3T3-E1 cells transfected with siLRP5; (**B**) Representative pictures of colonies of MC3T3-E1 cells transfected with siLRP5; (**C**) The clonogenic potential of MC3T3-E1 cells transfected with siLRP5; (**D**) Expression levels of RUNX2 in MC3T3-E1 cells upon siLRP5 treatment; (**E**) Fold changes of sclerostin, Hey1, Smad6, and Smad7 in MC3T3-E1 cells upon siLRP5 treatment. * *p* < 0.05, ** *p* < 0.01. *n* = 3/group.

Since our data showed that the miR-9-5p suppressed the GSK3β, a component of the Wnt signaling pathways. We further studied their upstream regulator LRP5, an important co-receptor in the Wnt pathways. We seeded the same numbers of MC3T3-E1 cells (500 cells/well) in six-well plates. The cells were transfected with a serial of silence RNA siLRP5 (15, 30, or 60 nM) to silence the function of LRP5. Every group contained triplicate wells. Photos were taken five days after transfection. From Figure 6A we can see that the numbers of cells decreased in a dose-dependent manner, indicating that LRP5 were essential for cell proliferation. To further verify this point, we stained the cells with crystal violet. The results revealed that clonogenic survival were decreased in a dose-dependent manner following silence of LRP5 in the MC3T3-E1 cells (Figure 6B,C).

To further study the whether LRP5 played positive roles in skeletal development, we detected the levels of RUNX2 in MC3T3-E1 cells transfected with siLRP5 (5 nM). Results showed that the mRNA expression of RUNX2 decreased after the function of LRP5 was blocked by siLRP5 (Figure 6D). To confirm the positive effects of LRP5, we checked if LRP5 knockdown induced increased expression of negative regulators of skeletal development. According to previous studies [7,8,9,10,11], we detected four negative regulators, namely sclerostin, Hey1, Smad6, and Smad7. Results showed that all four negative regulators increased after the MC3T3-E1 cells were treated with siLRP5 (Figure 6E). Specifically, silence of LRP5 led to significantly increased sclerostin with fold change value of 8.5. These four increased negative factors, together with decreased LRP5, adversely accelerated the damage of bone formation.

Taken together, these results strongly suggested that LRP5 were essential for skeletal development.

## 3. Discussion

**Figure 7:**
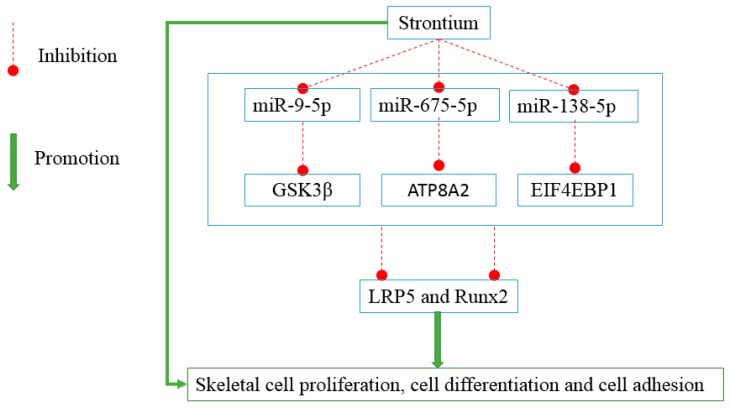
The flowchart of the main study objects and their relationships.

In conclusion, our results showed strontium decreased the expression levels of miR-9-5p, miR-675-5p, and miR-138-5p, and these three miRNAs inhibited the levels of LRP5 and RUNX2. These results indicate that strontium may activate the Wnt signaling pathways including LRP5 and then increase the levels of RUNX2 leading to osteoblast proliferation and differentiation by inhibiting these three miRNAs. In the current study we have revealed that GSK3β, ATP8A2, and EIF4EBP1 are the target genes of miR-9-5p, miR-675-5p, and miR-138-5p through three aspects. Firstly, target prediction tools have shown the exact binding sites between the miRNAs and the 3’UTR of the genes. Secondly, the downregulation of the target genes upon treatment of miRNAs indicates that the miRNAs have bound to the 3’UTR of the genes and degraded their target genes. Thirdly, the decrease of luciferase activity verified this point. The relationships found or verified in this study are summarized in the flowchart (Figure 7).

Our results showed that the expression levels of miR-370, miR-378, and miR-320 were inhibited by strontium. The enforced expression of miR-370 in MC3T3-E1 cells or stable transfection of MC3T3-E1 cells with miR-378 attenuated pre-osteoblast differentiation [4,22], indicating that strontium might promote cell differentiation by inhibiting the expression of miR-370 and miR-378. Concordant with our results, stable expression of miR-320 suppressed osteogenic differentiation [23].

Clonogenic assay showed that miR-9-5p inhibited the cell proliferation more significantly than the other two miRNAs and qPCR revealed miR-9-5p suppressed the expression levels of LRP5 and RUNX2 more significantly than the other two miRNAs, so these two methods generated concordant results, that is to say, miR-9-5p more adversely impaired the cell proliferation and differentiation. Our results showed that miR-138-5p impaired skeletal development. Martin *et al.* [15] reported that miR-138-5p had anti-osteoblastic effects, supporting our results.

In the clonogenic assay we used final concentration 5 nM miR-9-5p, 10 nM miR-675-5p, and 10 nM miR-138-5p. The concentration of miRNAs may influence the colony formation rate of the cells. Excessively high concentration of miRNAs may slow the cell proliferation. Our results have shown that 5 nM miR-9-5p almost killed all the cells. In fact, we also used 20 nM miR-9-5p and the results were similar with that of 5 nM, indicating that 5 nM miR-9-5p is enough to inhibit cell proliferation and kill the cells. Recent studies reported that miR-9 could mediate the cell apoptosis in ischemic stroke [24].

Wnt signaling pathways played important roles in osteoblast differentiation and bone formation [25]. Since LRP5 was a critical co-receptor of Wnt signaling pathways, its loss of function may limit the osteoblast cell proliferation and differentiation, which was supported by our data.

miR-9 restoration retarded HCC cell proliferation and migration. Since our results have shown that miR-9 inhibits MC3T3-E1 cell proliferation, these results indicate that miR-9 inhibits cell proliferation not only in bone cells, but also in cancer cells, thus providing potential therapeutic targets and novel prognostic biomarkers. miR-675 was upregulated in livers with HBV infection and was associated with immune response pathways [26], indicating that viruses and drugs affect the cells and the bodies through altering the levels of miRNAs such as miR-675. Further studies of these miRNAs are needed to reveal the mechanisms of drugs and disease progression.

## 4. Experimental Section

### 4.1. Cell Cultures and Reagents

MC3T3-E1 cells were cultured in Alpha MEM medium (Gibco, Hong Kong, China) supplemented with 10% FBS (Gibco, Hong Kong, China), penicillin, streptomycin sulfate, and fungizone. ATDC5 cells were cultured in DMEM/F12 supplemented with 10% FBS, penicillin, streptomycin sulfate, and fungizone. Strontium ranelate was purchased from Servier (Orléans, France). The sequences of miRNA mimics (Qiagen, Duesseldorf, Germany) were as follows: miR-9-5p: 5′-UCUUUGGUUAUCUAGCUGUAUGA-3′; miR-675-5p: 5′-UGGUGCGGAAAGGGCCCACAGU-3′; miR-138-5p: 5′-AGCUGGUGUUGUGAAUCAGGCCG-3′.

### 4.2. Plasmid Construction

To generate the 3’UTR of GSK3β containing the binding sites of miR-9-5p ligating luciferase plasmid, we extracted RNA from the cells and then converted the RNA into cDNA. Using the cDNA as the template, we amplified the fragment containing the binding sites of the miR-9-5p by using Platinum^®^ Pfx DNA Polymerase (Invitrogen, Hong Kong, China). We used the software CLC Genomics Workbench to design primers, which were as follows: Forward 5’-CGAGCTCTGTGTGTGTGTGTGTGTG-3’; Reverse: 5’-TTCCGCGGCCGCTATGGCCGACGTCGACGGGAATGGGGAAAGGGAA-3’. After confirmation of electrophoresis, the PCR products were purified, digested with SacI and SalI, purified, ligated with the vector pmirGLO Dual-Luciferase miRNA Target Expression Vector (Promega Corporation, Madison, WI, USA), followed by the transformation by heat shock, analyzing the transformants by colony PCR, electrophoresis, sequencing, and plasmid extraction.

The 3′UTR of ATP8A2 and EIF4EBP1 containing the binding sites of miR-675-5p and miR-138-5p were synthesized. The sequences for 3′UTR of ATP8A2 and EIF4EBP1 were as follows: ATP8A2: 5′-CCCUGGAAGAGUCUUCCGCACCC-3′; EIF4EBP1: 5′-CCUCCUCUUCGUGAACACCAGCA-3′. We dissolve the oligonucleotides in water to 20 µM and set up total 20 µL reactions as follows: 1 µL forward oligo, 1 µL reverse oligo and 18 µL 10 mM Tris-HCl, pH 8.5 or 8.0. The mixture was heated to 95 °C for 2 min and then cooled to room temperature. The inserts 3′UTR of ATP8A2 and EIF4EBP1 were cloned into pMIR-REPORT Luciferase vector (Applied Biosystems, Hong Kong, China).

### 4.3. Transfection

For miRNA transfection and siLRP5 transfection, we added 0.5% HiPerfect Transfection Reagent (Qiagen) into serum-free medium. The mixture of medium without serum and miRNAs was incubated for 10 min at room temperature before adding them into cells. For plasmid transfection, MC3T3-E1 cells were seeded in 96 well plates. Cells were co-transfected with 60 ng/well of plasmids and miRNA mimics by using Lipofectamine 2000 (Invitrogen). For siRNA, RNA extraction from the cells was conducted at 48 h after the transfection. For miRNAs, RNA extraction was conducted at 72 or 48 h after transfection.

### 4.4. Clonogenic Assay (Colony Formation Assay)

Clonogenic assay was conducted according to the published protocol [27]. In brief, MC3T3-E1 cells were seeded with the density of 500 cells/well of the six-well plate. miR-9-5p, miR-675-5p, and miR-138-5p were transfected and their concentrations were maintained by adding these miRNAs three days after the first transfection. One week after the first transfection, the cells were fixed by 70% ethanol and stained by 0.5% crystal violet (Sigma, St. Louis, MO, USA). Clones were considered to represent viable cells if they contained in excess of 50 cells.

### 4.5. Cell Viability Assay

Cell viability assay was conducted by using CellTiter-Blue^®^ Cell Viability Assay (Promega Corporation) using resazurin. In brief, the MC3T3-E1 and ATDC5 cells were seeded with a concentration of 100 cells/150 μL and transfected with a serial dilution of miRNAs (500, 250, 125, 62.5, and 31.25 nM). The concentration were maintained by a second transfection three days after the first transfection. Six days after the first transfection, the cells were added with CellTiter-Blue^®^ Reagent and the fluorescence was measured.

### 4.6. Cell Cytotoxicity Assay

We test the cell cytotoxicity of miR-9-5p by using colorimetric assay (WST-1 based). In brief, MC3T3-E1 cells were seeded with the concentration of 5000 cells/well in the 96-well plate and transfected with a serial dilution of miR-9-5p (0, 200, 400, and 800 nM). The assay were conducted after two days. The wavelength for measuring the absorbance of the formazan product was 440 nm and reference wavelength was 690 nm. The blank background control was made by adding the same volume of culture medium and WST-1 into one well.

### 4.7. TUNEL Cell Apoptosis Assay

We conducted cell apoptosis assay by using ApopTag^®^ Red In Situ Apoptosis Detection Kit (Merck Millipore Corporation, Darmstadt, Germany), which detected apoptosis by indirect TUNEL method, utilizing an anti-digoxigenin antibody with a rhodamine fluorochrome. In brief, the MC3T3-E1 were seeded into the chamber slides (8000 cells/150 µL/well) and transfected with three miRNAs (500 nM). After two days, the assay was conducted according to manufacturer’s standard manual. The cells were fixed in 1% paraformaldehyde in PBS and post-fixed in ethanol: acetic acid (2:1), sequentially applied with Equilibration Buffer, TdT Enzyme, Stop/Wash Buffer, Anti-Digoxigenin Conjugate and Rhodamine/DAPI Staining. When taking photos, we assured consistent parameters including scale, exposure time and analog gain of the fluorescence microscopy for each picture so that all the pictures were comparable.

### 4.8. Animal

All procedures were approved by the Committee on the Use of Live Animals in Teaching and Research of The University of Hong Kong. Animal licenses were approved by the Department of Health of the Government of Hong Kong. Mice with a B6.129P2 background were imported from Jackson Laboratory (Bar Harbor, ME, USA). The heterozygous mice were crossed to obtain LRP5 knockout mice. For genotypes analysis of the mice, DNA extraction were conducted by using the ears of the mice, followed by PCR and agarose gel electrophoresis. The three primers used in the PCR were as follows: 5′-CACTGCATGGATGCCAGTGAGGTGG-3′; 5′-GCTGCCACTCATGGAGCCTTTATGC-3′; 5′-CGCTACCGGTGGATGTGGAATGTGT-3′. We treated the mice with strontium ranelate daily by oral gavage. The strontium ranelate was dissolved in water and made into suspension. The dosage was 33.3 ng drug powder per gram (body weight).

### 4.9. RNA Extraction

Total RNA from the cells or the bone of the mice was extracted by using TRIzol. The femur, tibia, and fibula were cleaned of soft tissue and used for RNA extraction. For every well of six-well plate, we used 1 mL TRIzol for RNA extraction from the cells. For RNA extraction from the bone, we used 1 mL TRIzol for approximately 200 mg fresh bone.

### 4.10. Poly (A) Polymerase Tailing

For expression of miRNAs detection, the RNA was polyadenylated by Poly (A) Polymerase Tailing Kit (Epicentre^®^ Biotechnologies) before reverse transcription.

### 4.11. Reverse Transcription

The RNA was mixed with random primer and reverse transcription was performed by using the High Capacity cDNA Reverse Transcription Kit (Applied Biosystems). Diluted cDNA was used for PCR. For expression of miRNAs detection, the polyadenylated RNA was mixed with reverse transcription primer (sequence: 5′-GCGAGCACAGAATTAATACGACTCACTATAGGTTTTTTTTTTAG-3′) instead of random primer.

### 4.12. Quantitative Real-Time PCR (qPCR)

qPCR was performed using the SYBR Green PCR Master Mix (Life Technologies Limited, Hong Kong, China) on a 7900HT Fast Real-Time PCR System (Applied Biosystems). Primers for qPCR were purchased from the Integrated DNA Technologies. miRNA detection was conducted according to the published paper [28]. In brief, the reaction was incubated at 95 °C for 10 min, followed by 45 cycles of 95 °C for 10 s and 60 °C for 1 min. For melting curve analysis the reactions were heated to 95 °C for 5 s and 60 °C for 15 s, then cooled to 40 °C. For gene detection, the reaction was incubated at 95 °C for 10 min followed by 40 cycles of 95 °C for 15 s and 60 °C for 1 min. Reverse primer for all the miRNAs were called RACE. The sequences for RACE, all the forward primers of miRNAs, and all the primers of genes were listed in Table 1.

**Table 1:** Sequences of all primers.

Primer Name	Sequences (5′–3′)
RACE Reverse	GCGAGCACAGAATTAATACGAC
miR-9-5p Forward	GCGTCTTTGGTTATCTAGCTGTA
miR-675-5p Forward	TGGTGCGGAAAGGGCCCACAGT
miR-138-5p Forward	AGCTGGTGTTGTGAATCAGGCCG
miR-370 Forward	GCCTGCTGGGGTGGAACCTGGT
miR-23a Forward	ATCACATTGCCAGGGATTTCC
miR-103 Forward	ACACTCCAGCTGGGAGCAGCATTGTAC
GAPDH Forward	ATTGTCAGCAATGCATCCTG
GAPDH Reverse	ATGGACTGTGGTCATGAGCC
ALP Forward	AACCCAGACACAAGCATTCC
ALP Reverse	GCCTTTGAGGTTTTTGGTCA
COL1A1 Forward	GCCAAGAAGACATCCCTGAA
COL1A1 Reverse	GCCATTGTGGCAGATACAGA
RUNX2 Forward	AAGTGCGGTGCAAACTTTCT
RUNX2 Reverse	ACGCCATAGTCCCTCCTTTT
GSK3β Forward	TTGGACAAAGGTCTTCCGGC
GSK3β Reverse	AAGAGTGCAGGTGTGTCTCG
ATP8A2 Forward	ACGAGGGACGTGCTCATGAAGC
ATP8A2 Reverse	CCTCAAGTGTCACCAGCAGGCT
EIF4EBP1 Forward	CTAGCCCTACCAGCGATGAG
EIF4EBP1 Reverse	CCTGGTATGAGGCCTGAATG
LRP5 Forward	TGCCACTGGTGAGATTGAC
LRP5 Reverse	ACTGCTGCTTGATGAGGAC
Cyclin-D1 Forward	GCGTACCCTGACACCAATCT
Cyclin-D1 Reverse	CTCCTCTTCGCACTTCTGCT
CyclinA Forward	CAGAGGCCGAAGACGAGAC
CyclinA Reverse	TCAGCTGGCTTCTTCTGAGC
CyclinE Forward	GTTATAAGGGAGACGGGGAG
CyclinE Reverse	TGCTCTGCTTCTTACCGCTC
PCNA Forward	TTTGAGGCACGCCTGATCC
PCNA Reverse	GGAGACGTGAGACGAGTCCAT
Integrin α2 Forward	AAGTGCCCTGTGGACCTACCCA
Integrin α2 Reverse	TGGTGAGGGTCAATCCCAGGCT
Integrin α5 Forward	ACCACCTGCAGAAACGAGAGGC
Integrin α5 Reverse	TGGCCCAAACTCACAGCGCA
Integrin Αv Forward	TCCCACCGCAGGCTGACTTCAT
Integrin Αv Reverse	TCGGGTTTCCAAGGTCGCACAC
Integrin β1 Forward	TTCAGACTTCCGCATTGGCT
Integrin β1 Reverse	AATGGGCTGGTGCAGTTTTG
Fibronectin Forward	TGCAGTGGCTGAAGTCGCAAGG
Fibronectin Reverse	GGGCTCCCCGTTTGAATTGCCA
FAK Forward	AGCACCTGGCCACCTAAGCAAC
FAK Reverse	CATTGGACCGGTCAAGGTTGGCA
Vinculin Forward	TCAAGCTGTTGGCAGTAGCCGC
Vinculin Reverse	TCTCTGCTGTGGCTCCAAGCCT
Osteocalcin Forward	AGCAGGAGGGCAATAAGGTAGT
Osteocalcin Reverse	TCGTCACAAGCAGGGTTAAGC
Osteonectin Forward	ATGTCCTGGTCACCTTGTACGA
Osteonectin Reverse	TCCAGGCGCTTCTCATTCTCAT
Osteopontin Forward	TGATTCTGGCAGCTCAGAGGA
Osteopontin Reverse	CATTCTGTGGCGCAAGGAGATT
Type I collagen Forward	CTCCTGACGCATGGCCAAGAA
Type I collagen Reverse	TCAAGCATACCTCGGGTTTCCA
Type III collagen Forward	CCCTGGCTCAAATGGCTCACCA
Type III collagen Reverse	CCTTTCCACCAGGACTGCCGTT
Cadherin 11 Forward	GGCCCAAACAGGTATCATCA
Cadherin 11 Reverse	TTGGTTGTCCCTGAGAGTCC
MAPK1 Forward	ATCCGGGCACCAACCATTGAGC
MAPK1 Reverse	GTGGTCATTGCTGAGGTGCTGTGT
NFkB Forward	AACCCATCGCCTTGGCATCCAC
NFkB Reverse	AGTCGAAAAGGGCGTTGGCGT
FasL Forward	GAGAATTGCTGAAGACATGACAATCC
FasL Reverse	ATGGCTGGAACTGAGGTAGTTTTCAC
p53 Forward	TAACAGTTCCTGCATGGGCGGC
p53 Reverse	CGGAGGCCCATCCTCACCATCATCA
HEY1 Forward	GCATACGGCAGGAGGGAAA
HEY1 Reverse	CTGGGAAGCGTAGTTGTTGAGAT
Sost Forward	GGAATGATGCCACAGAGGTCAT
Sost Reverse	CCCGGTTCATGGTCTGGTT
Smad7 Forward	GGACGCTGTTGGTACACAAG
Smad7 Reverse	GCTGCATAAACTCGTGGTCATTG
SMAD6 Forward	GCTCTAGGAATGCAGACGCTG
SMAD6 Reverse	CAACAGGCAGTCAGCACAGTC

### 4.13. Luciferase Reporter Assay

Firefly luciferase activities were measured 48 h after transfection by using dual-light luminescent reporter gene assay kit (Applied Biosystems) according to the standard protocol. In brief, cell lysate was mixed with reagents for the luciferase reaction, the luciferase signal was measured by using a VICTOR™ X3 Multilabel Plate Reader (PerkinElmer, Waltham, MA, USA). After 40 min incubation, β-galactosidase signal was initiated by addition of Accelerator-II and detected.

### 4.14. Statistical Analysis

Statistical analysis was performed using a *t*-test, and *p* < 0.05 was considered statistically significant.

## 5. Conclusions

miR-9-5p, miR-675-5p, and miR-138-5p damage strontium and LRP5-mediated skeletal cell proliferation, differentiation, and adhesion, and induce cell apoptosis by targeting GSK3β, ATP8A2, and EIF4EBP1, respectively.

## Supplementary Materials

Supplementary materials can be found at http://www.mdpi.com/1422-0067/17/2/236/s1.
