# Supplementary material for: miR-9-5p, miR-675-5p and miR-138-5p Damages the Strontium and LRP5-Mediated Skeletal Cell Proliferation, Differentiation, and Adhesion
Source: Int J Mol Sci. 2016 Feb 15;17(2):236. doi: 10.3390/ijms17020236 (PMC4783967; doi:10.3390/ijms17020236)
Supplement: Supplementary file 1 [file ijms-17-00236-s001.pdf]

Supplementary Materials: miR-9-5p, miR-675-5p, and miR-138-5p Damages the Strontium and LRP5-Mediated Skeletal Cell Proliferation, Differentiation, and Adhesion

Tianhao Sun, Frankie Leung and William W. Lu

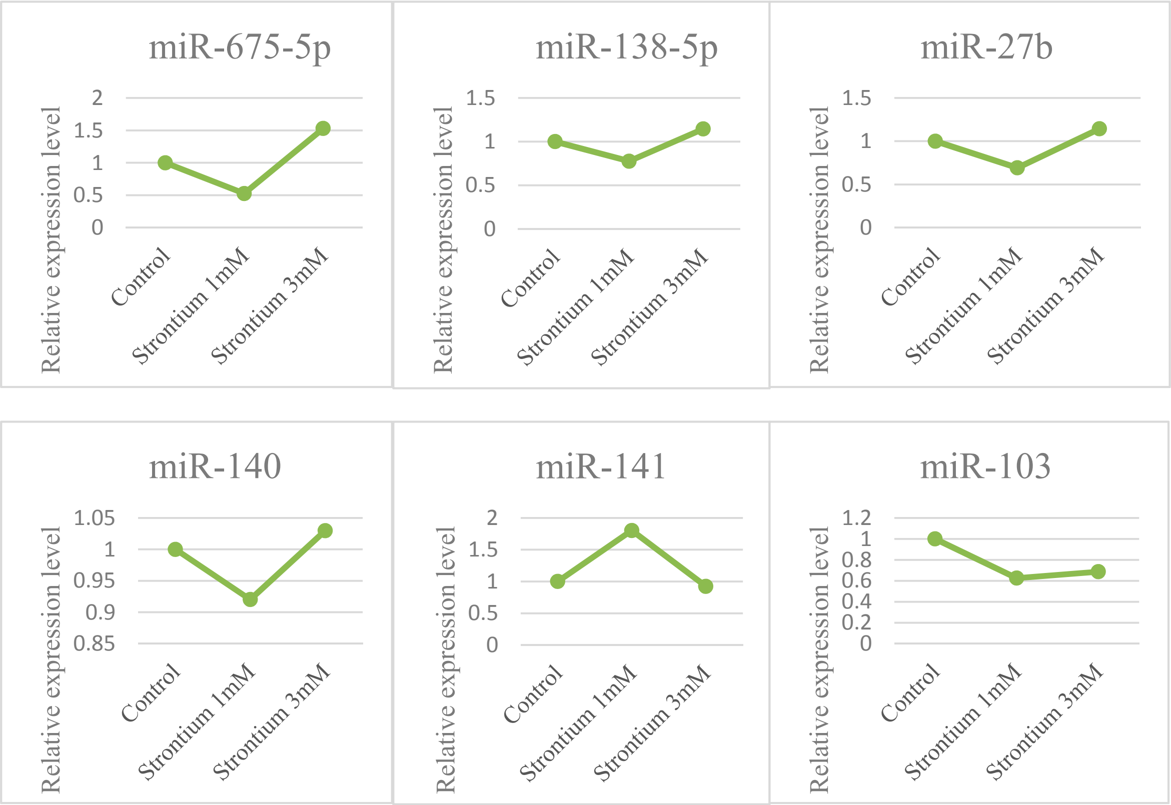

Figure S1. Expression levels of miRNAs in MC3T3-E1 cells upon strontium ranelate treatment.

Table S1. The expression changes of the 15 cell adhesion makers of MC3T3-E1 cell treated with miR-9-5p, miR-675-5p, and miR-138-5p mimics.

Name	miR-9-5p			miR-675-5p			miR-138-5p		
	Average Fold Change	*p* Value	SD	Average Fold Change	*p* Value	SD	Average Fold Change	*p* Value	SD
Integrin α2	0.44	0.004	0.17	0.65	0.214	0.41	0.50	0.163	0.51
Integrin α5	1.07	0.544	0.19	0.31	0.000	0.05	0.36	0.000	0.03
Integrin αV	0.54	0.001	0.09	0.24	0.000	0.10	0.23	0.000	0.04
Integrin β1	0.81	0.144	0.18	0.52	0.041	0.28	0.51	0.007	0.16
Fibronectin	0.63	0.001	0.07	0.37	0.001	0.13	0.46	0.003	0.15
FAK	1.26	0.278	0.36	0.54	0.037	0.26	0.48	0.005	0.16
Vinculin	0.45	0.001	0.10	0.35	0.005	0.20	0.41	0.000	0.09
Osteocalcin	1.10	0.889	1.20	0.47	0.003	0.14	0.80	0.143	0.19
Osteonectin	0.55	0.005	0.14	0.76	0.391	0.43	0.95	0.785	0.30
Osteopontin	2.60	0.017	0.70	0.89	0.706	0.47	1.22	0.364	0.37
Type I collagen	0.35	0.000	0.05	0.20	0.000	0.04	0.22	0.000	0.05
Type III collagen	0.23	0.000	0.03	1.17	0.038	0.10	0.85	0.073	0.11
Cadherin 11	0.74	0.054	0.17	0.33	0.002	0.17	0.60	0.005	0.13
MAPK	1.13	0.480	0.29	0.29	0.002	0.16	0.43	0.002	0.13
NFKB	0.74	0.013	0.11	0.39	0.000	0.07	0.72	0.028	0.14
